# Genome-Wide Association Identifies *TBX5* as Candidate Gene for Osteochondrosis Providing a Functional Link to Cartilage Perfusion as Initial Factor

**DOI:** 10.3389/fgene.2013.00078

**Published:** 2013-05-10

**Authors:** Noppawan Rangkasenee, Eduard Murani, Ronald M. Brunner, Karl Schellander, Mehmet Ulas Cinar, Henning Luther, Andreas Hofer, Monika Stoll, Anika Witten, Siriluck Ponsuksili, Klaus Wimmers

**Affiliations:** ^1^Institute for Genome Biology, Leibniz Institute for Farm Animal BiologyDummerstorf, Germany; ^2^Animal Breeding and Husbandry Group, Institute of Animal Science, University of BonnBonn, Germany; ^3^SUISAG, AllmendSempach, Switzerland; ^4^Leibniz Institute for Arteriosclerosis ResearchMuenster, Germany

**Keywords:** osteochondrosis, polymorphism, genome-wide association, leg weakness, epistatis, pigs

## Abstract

Osteochondrosis (OC) is an orthopedic syndrome of the joints that occurs in children and adolescents and domestic animals, particularly pigs, horses, and dogs. OC is the most frequent cause of leg weakness in rapidly growing pigs causing animal welfare issues and economic losses. In this study, a genome-wide association study (GWAS) was performed using the Porcine 60k SNPChip in animals of the breed Large White (*n* = 298) to identify chromosome regions and candidate genes associated with OC lesion scores. A total of 19 SNPs on chromosomes (SSC) 3, 5, 8, 10, 14, and 18 were significantly associated with OC lesion scores (*p*-values ≤ 10^−5^). The SNPs *MARC0098684*, *MARC00840086*, *MARC0093124*, and *ASGA0062794* at SSC14 36.1–38.2 Mb encompass a region of six linkage disequilibrium (LD) blocks. The most significant SNP *ASGA0062794* is located in a LD block spanning 465 kb and covering the gene encoding T-box transcription factor 5 (*TBX5*). A SNP (c.54T > C) identified in *TBX5* was significantly associated with OC lesion scores in a single-marker analysis. *TBX5* c.54T > C showed highest LD with *ASGA00627974* (*r*^2^ = 0.96) and superior association with OC lesion scores over other SNPs when included in the genome scan, whereas its treatment as an additional fixed effect in the GWAS statistical model led to a drop of significance of nearby markers. Moreover, real-time PCR showed different transcript abundance of *TBX5* in healthy and defect cartilage. The results imply that the association signal obtained on SCC14 is largely attributable to *TBX5* c.54T > C likely to be in LD with a regulatory polymorphism of *TBX5*. The transcription factor *TBX5* interacts with *GJA5* and *MEF2C* both being involved in vascularization. This study provides evidence for epistatic interaction of *TBX5* and *MEF2C*, thus supporting deficiency of blood supply to growth cartilage as being fundamental for the initiation of OC.

## Introduction

Osteochondrosis (OC) is a joint disorder characterized by the disturbance of endochondral ossification in the articular cartilage and the epiphyseal growth plates that is common in human beings and several animal species including pigs (Crenshaw, 2006; Ytrehus et al., 2007). OC is regarded as a major cause of a syndrome called leg weakness in pigs (Reiland et al., 1978; Lundeheim, 1987; Jørgensen et al., 1995; Kirk et al., 2008), that affects animal welfare and longevity and causes the culling of breeding pigs, resulting in economic losses (Yazdi et al., 2000; Fukawa and Kusuhara, 2001). The selection for production traits, such as high growth capacity of skeletal muscles are suspected to predispose for pathogenic disorders of the osteochondrotic system (Uhlhorn et al., 1995). There are mismatches between the developmental maturation of the skeletal system and the direction and magnitude of muscle force on articular cartilage, epiphyseal plates, and subchondral bones (Uhlhorn et al., 1995; Bickhardt, 1998; Arnbjerg, 2007). The OC lesions mainly occur at the distal epiphysis of ulna, the medial condyles of humerus and femur (Nakano et al., 1987).

The etiology of OC is multifactorial including rapid growth, anatomic conformation, trauma, and dietary imbalances that promote formation of a fragile cartilage, failure of chondrocyte differentiation, subchondral bone necrosis, and failure of blood supply to the growth cartilage (Ytrehus et al., 2007). Moreover genetic disposition plays an important role in OC development (Kadarmideen et al., 2004; Ytrehus et al., 2007). The heritability of OC or leg weakness was estimated to be between 0.1 and 0.5 in different pig breeds (Reiland et al., 1978; Lundeheim, 1987; Jørgensen and Andersen, 2000). A number of quantitative trait loci (QTL) studies have identified chromosomal regions, which are involved in OC and/or traits related to leg weakness in pigs (Andersson-Eklund et al., 2000; Lee et al., 2003; Christensen et al., 2010; Uemoto et al., 2010). In addition, several candidate genes have been addressed and located within some of these QTL regions in different breed of pigs (Onteru et al., 2008; Fan et al., 2009).

In general linkage studies based on genotyping a limited number of highly polymorphic markers, mostly microsatellites, in special family designs have revealed numerous QTL regions for various traits (August 10, 2012, there have been 6,818 pig QTLs curated into the Pig QTLdb^1^, however, usually at low resolution. The development of a porcine 60k SNPchip (PorcineSNP60 BeadChip, Illumina) enabling simultaneous genotyping at several thousands of SNP distributed over the whole genome offer the opportunity of conducting genome-wide association studies (GWAS). GWAS provide a powerful approach for annotating phenotypic effects or mapping QTL of economically important traits in livestock, and it can be conducted using animals from commercial breeding schemes and potentially deliver trait-associated markers with strong linkage disequilibrium (LD) to causal polymorphisms. So far, published GWAS in pigs address fatness, immune, and reproductive traits (Duijvesteijn et al., 2010; Ponsuksili et al., 2011; Uimari et al., 2011; Boddicker et al., 2012; Onteru et al., 2012). Recently, a GWAS identified several candidate genes for structural soundness traits in pigs (Fan et al., 2009). In this study, a GWAS was performed with the Illumina Porcine SNP60 BeadChip in a commercial Large White population aiming at the detection of chromosome regions and candidate genes associated with OC lesion scores. Furthermore, we confirmed that OC lesion scores are associated with a polymorphism of the *T-box transcription factor 5* (*TBX5)* gene which represents a functional and according to the GWAS, also a positional candidate gene for OC.

## Materials and Methods

### Animals and phenotypes

Genomic DNA and phenotypic data were obtained from animals of the breed Large White (*n* = 298) from SUISAG during 2005–2008. The animals used were selected from the two ends of the distribution of OC lesion scores (OCsum ≤ 8 and OCsum ≥ 12, see below) recorded from 2,622 animals. In order to avoid bias due to any obvious parameter that might affect OC scores, selection was done accounting for sex, year, body weight, and ancestry; in fact, selected animals represent offspring of 141 boars and 264 dams. The animals were kept in the performance test station of SUISAG and fed *ad libitum* during the whole testing period from 30 to at average 103 kg of body weight. The animals were examined for osteochondral lesions at seven positions of the carcass after dissection (Luther et al., 2007). In brief, for lesion scoring cartilage of the head of humerus (HH), medial part of condylus humeri (CMH) (trochlea humeri), lateral part of condylus humeri (CLH) (capitulum humeri), proximal at radius and ulna (RUP), head of femur (HF), condylus medialis femoris (CMF), and the distal epiphyseal cartilage of ulna (DEU) of the left carcass half were inspected by a trained person. After examination of the surface and shape of the bones and joints *in situ*, each bone was longitudinally sawed, and the state and shape of the cartilage (thickening of the cartilage, subchondral lesions, irregular osteochondral junction or fissures at the osteochondral junction, macroscopic defects of the surface) and the epiphyses was examined at the cutting surface. Scores from 1 to 4 were defined (score 1 = “no visible osteochondral lesion,” scores 2–4 denote “mildly to severely affected,” respectively). Osteochondral lesions at the DEU and CMF are scored from 1 to 6, because these lesions were more variable (Luther et al., 2007). In addition to the scores of OC lesions at distinct sides, scores of all joints were summed to give an OC lesion score sum (OCsum), and each animal was assigned to either the group OCcat high (*n* = 136) or OCcat low (*n* = 162) (corresponding to OCsum ≥ 12 or ≤ 8, respectively).

For quantification of transcript abundance of *TBX5* articular cartilage samples were obtained from a Duroc × Pietrain crossbreed population where OC lesions were monitored histopathologically, as previously described. Therefore, slices of the cartilage surface of CMF covering macroscopically non-affected areas and, if present, affected regions were stored at −80°C. For lesion scoring, histological sections of 7 μm were prepared and stained in duplicate after fixation and demineralization. Representative section were scored on a scale from 1 (=normal) to 4 (=severe) (score 1 denotes homogenous cartilage matrix; columns of chondrocytes visible, score 2 means homogenous cartilage matrix; low-grade fraying of the surface, score 3 shows surface erosions, fraying in deeper cartilage layers, score 4 means surface erosions, fraying in deeper cartilage layers and cavitations) (Laenoi et al., 2010; Rangkasenee et al., 2013). Finally, four divergent sib pairs with low (1) and high (4) histologic OC lesion scores were selected for RNA preparation and subsequent real-time RT-PCR.

### Genotyping

Animals were genotyped using the PorcineSNP60 BeadChip (Illumina, Inc., San Diego, CA, USA) and approved standard techniques as recommended by the manufacturer. This array contains 62,163 SNPs distributed throughout the porcine genome. Raw data were normalized and assigned a cluster position for genotyping with the GenomeStudio software (Illumina, Inc.). SNPs were filtered with call rates >99%. Markers were excluded if they had low minor allele frequency <5%. Deviation from Hardy-Weinberg equilibrium and the degree of LD were analyzed using HaploView 4.2 (Barrett et al., 2005). LD was estimated for the chromosomal regions where multiple candidate SNPs were located. Finally, 48,716 SNPs remained for the GWAS.

Two genes, namely T-box transcription factor 3 (*TBX3*) and T-box transcription factor 5 (*TBX5)* both located in close proximity to each other and a number of significant markers, were screened for SNPs in the coding region by comparative sequencing of PCR fragments of eight individuals. The PCR products were purified and comparatively sequenced in both directions using an ABI3130 DNA Analyzer. We detected a single SNP at g.38,175,026T > C or position 54 relative to the start codon (c.54T > C) of *TBX5*. Then, *TBX5* genotyping was performed by PCR-RFLP technique using the restriction enzyme *Pfe*I. The following primers were used: forward 5′-AAACTGCTGGTGGAAGCCTA-3′ and reverse 5′-GCAGAGAAAGGTGGGATTGA-3′. The PCR was performed in 20 μl volume containing 50 μg of genomic DNA, 1 × PCR buffer (with 1.5 mM MgCl_2_), 0.25 mM of dNTP, 0.2 μM of each primer, and 0.5 U of Taq DNA polymerase (GeneCraft, Germany). The PCR procedures were performed under the following conditions: initial denaturing at 95°C for 5 min followed by 40 cycles of 30 s at 95°C, 30 s at 62°C, and 1 min at 72°C, and final elongation of 10 min at 72°C. After PCR amplification, PCR products were checked for specific amplification on 1.5% agarose gels (Sigma-Aldrich, Taufkirchen) and digested by *Pfe*I enzyme. The digested products were separated using 3% agarose gels.

### Association analyses

**Figure A1:**
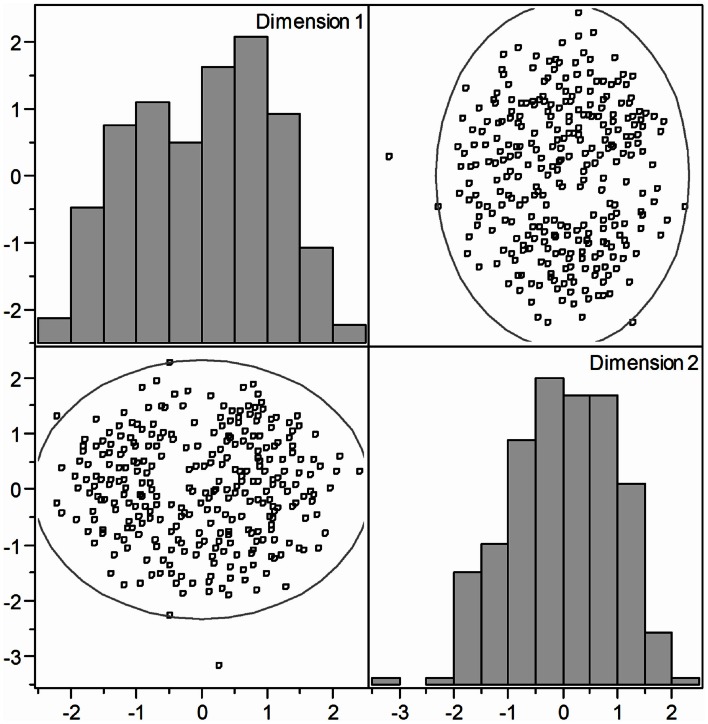
**Multidimensional scaling (MDS) analysis of an identity by state (IBS) of pig samples clustering showed that population stratification is not prominent**.

All statistical analyses were performed using JMP Genomics 5.0 (SAS Institute Inc, Cary, NC, USA). Although the subset of animals of a total of 2,622 animals was built in order to combat any bias due to population stratification, the success of these measures of the selection procedure in this regard was tested. Accordingly, to determine population stratification, pairwise clustering of identity by state (IBS) distance of the samples was computed using the IBS and multidimensional scaling (MDS) approach within JMP Genomics 5.0. MDS analysis show that all samples cluster together within indication of any subclusters, suggesting that population stratification is not prominent (Figure A1 in Appendix). Moreover, the genomic inflation factor (λ) was calculated according to Devlin and Roeder (1999). We obtained mild population stratification; corresponding genomic inflation factors based on median χ2 were λ = 1.18 for case-control and λ = 1.11 for the logistic regression.

We compared allele frequencies between case (OCcat high) and control (OCcat low) for testing trait association by a χ2 test with one degree of freedom. Whereas, for the GWA analysis to assess the association between SNPs and the ordinal traits of OC lesion scores, a logistic regression model (PROC LOGISTIC, JMP Genomics 5.0) was used with the fixed effect of line, sex, and slaughter weight as a covariate. Genotype effects were estimated as odds ratios (ORs) and 95% confidence intervals (95% CIs). ORs were estimated for homozygotes of the minor allele compared with homozygotes for the major allele and heterozygotes (dominant model). OR value less than 1 imply the desired effect of lower OC lesion scores. A significance threshold of *p*-value < 10^−5^ was used, equaling a false discovery rate (FDR) based *q-*value ≤ 0.1 (Storey, 2002; Storey et al., 2004). The same model was used to assess the effects of the SNP c.54T > C in *TBX5* on OC lesion scores. *p-Values*, ORs, and 95% CI were reported under an additive and dominant genetic model. LD block creation (JMP Genomics 5.0) was applied in order to define LD blocks across a 3.4 Mb region on chromosome 14. The *D*′ and *r^2^* coefficient (calculated by PROC ALLELE) were used to calculate pairwise LD estimates. Finally, LD measure *D*′ (calculated by PROC ALLELE) between pairs of SNPs was estimated to create blocks of consecutive SNPs based on those in strong LD (Gabriel et al., 2002).

### Identification of candidate genes

Gene annotations for 1 Mb genomic intervals around the significantly associated SNPs were identified by the Ensemble genome browser (Build9)^2^ (last accessed June 19, 2012) and the NCBI map viewer^3^ (last accessed June 19, 2012). The QTL location for relevant traits retrieved from PigQTL^4^ (last accessed June 19, 2012) was integrated, to obtain connections between QTL and candidate regions found from GWAS.

### Transcript quantification

Total RNA was isolated from the articular cartilage of CMF of four divergent sib pairs using TRI reagent (Sigma-Aldrich, Taufkirchen, Germany) according to manufacturer’s protocol. After DNase treatment a column-based purification using the RNeasy Mini Kit (Qiagen, Hilden, Germany) was done. The RNA samples were visualized on 1% agarose gels containing ethidium bromide to check RNA integrity. RNA was quantified by spectrometry with a NanoDrop ND-1000 spectrophotometer (PEQLAB, Erlangen, Germany). In addition, absence of DNA contamination was checked by using the RNA as a template in standard PCR amplifying fragments of the glyceraldehyde-3-phosphate-dehydrogenase (*GAPDH*) gene. All RNAs were stored at -80°C for further analysis.

RNA was reverse-transcribed into cDNA using random primers and an oligo dT primer in the presence of Superscript III reverse transcriptase (Invitrogen, Karlsruhe, Germany). Quantitative real-time RT-PCR (qPCR) was performed on LightCycler480 system using the LightCycler 480 SYBR Green I Master kit (Roche, Mannheim, Germany). Two common housekeeping genes *HPRT* and *RPL13A* were used for the data normalization. The primer sequences are shown in Table 1. The amplification was conducted in duplicate according to manufacturer’s instructions in a final volume of 10 μl using 5.0 μl of LightCycler 480 SYBR Green I Master mix (Roche), 2.0 μl of aqua dest., 10 μM (0.5 μl) of each primer, and 40 ng (2 μl) of cDNA. The temperature profiles comprised an initial denaturation step at 95°C for 10 min and 40 cycles consisting of denaturation at 95°C for 15 s, annealing at 60°C for 10 s and extension/fluorescence acquisition at 72°C for 15 s. For all assays threshold cycles were converted to copy numbers using a standard curve generated by amplifying serial dilutions of an external PCR standard (10^7^–10^2^ copies). The 2^−ΔCt^ and 2^−ΔΔCt^ methods were used to calculate relative expression of the target gene *TBX5*. For the 2^−ΔCt^ method, mean Ct values of *TBX5* in each sample was normalized to housekeeping gene values (Livak and Schmittgen, 2001). The 2^−ΔΔCt^ method was used to analyze changes in transcript abundance between normal (histological OC lesion score = 1) and affected (histological OC lesion score = 4) tissues, where the ΔCt values in the affected tissues was normalized to the ΔCt values in the normal tissues to obtain the ΔΔCt values. Statistical analysis was performed by mixed model analysis using Proc Mixed of SAS version 9.2 (SAS Instititute, Cary, NC, USA) with the fixed effects of sex and OC lesion score; family was used as a random effect and slaughter weight as covariate. Differences were considered significant at *p*-value ≤ 0.05.

**Table 1:** **Primers used for quantitative real-time polymerase chain reaction (qPCR)**.

Gene symbol	Gene name	Primer sequence (5′–3′)	Accession no.
*TBX5*	T-box transcript factor 5	F: gccgataataaatggtctgtg	XM_003132900.1
		R: tagaataatgtgcccaaacgg	
*HPRT*	Hypoxanthine phosphoribosyltransferase	F: gtgatagatccattcctatgactgtaga	NM_001032376.2
		R: tgagagatcatctccaccaattactt	
*RPL13A*	Ribosomal protein L13A	F: agaggaaggagaaggccaag	XM_003127305.1
		R: gaatccatgggtcttgagga	

## Results

The phenotype distribution of OC lesion scores at different joints among the animals used is shown in Table 2. The bimodal distribution of OC lesion scores of DEU is the result of sampling extreme phenotypes. Among the investigated regions, HH, HF, and RUP showed low incidence of high OC lesion scores, while the most affected regions were CMH, CMF, and DEU. Therefore, OC lesion scores of CMH, CMF, DEU, and OCcat were analyzed in this study.

**Table 2:** **Phenotype distribution of osteochondrosis (OC) lesion scores among the animals of the large white population**.

Traits (*n*)	OC lesion scores (*n*)					
	1	2	3	4	5	6
HH (298)	295	0	2	1		
HF (298)	297	1	0	0		
CMH (298)	239	31	28	0		
CMF (298)	188	87	21	2		
RUP (298)	273	25	0	0		
DEU (298)	31	133	15	47	70	2
	Low	High				
OCcat (298)	162	136				

*HH, head of humerus; HF, head of femur; CMH, medial part of condyles humeri; CMF, condylus medialis femoris; RUP, radius and ulna proximal; DEU, distal epiphyseal cartilage of ulna; OCsum, summary score of OC lesions; OCcat, category of animals with either high (OCsum ≥ 12) or low (OCsum ≤ 8) OCsum*.

**Figure 1:**
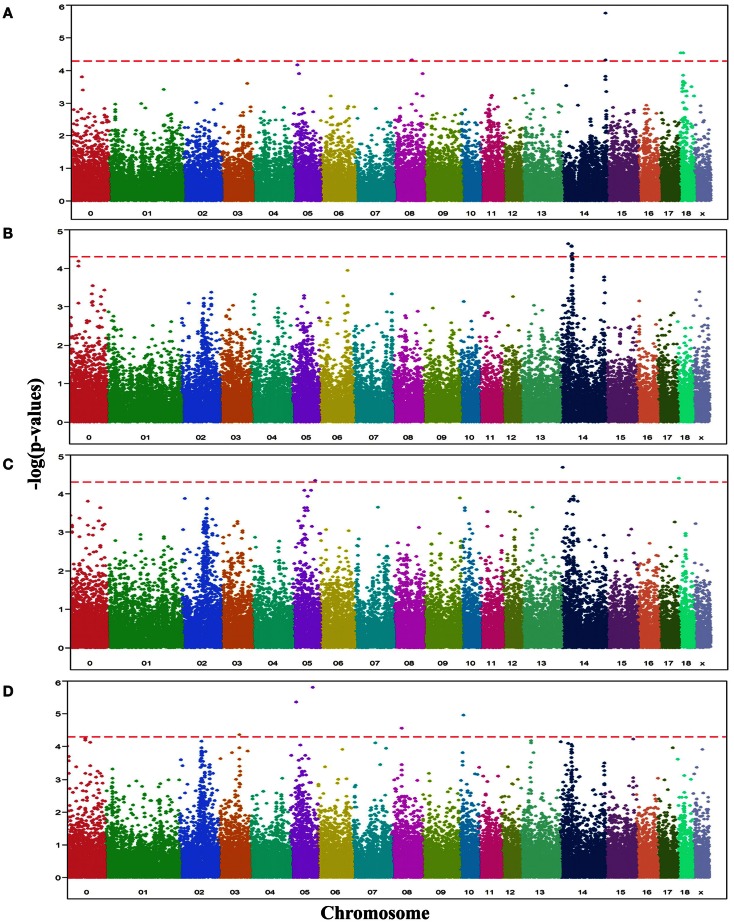
**Manhattan plots displaying the significance of associations of SNPs with OC lesion scores at (A) medial part of condylus humeri (CMH), (B) condylus medialis femoris (CMF), (C) distal epiphyseal cartilage of ulna (DEU), and (D) category of OC (OCcat)**. The *p*-values (−log10 transformed) on the vertical axis are plotted against the genomic position of SNPs on the horizontal axis. The horizontal dashed line indicates the genome-wide significance level (*q*-value ≤ 0.1).

Analyses of GWA revealed 19 SNPs that showed significant association (*p*-value ≤ 10^−5^, *q*-value ≤ 0.1) with either one or more of the OC lesion score traits. Significant SNPs were distributed along chromosomes (SSC) 3, 5, 8, 10, 14, and 18. The Manhattan plots of −log_10_(*p*-values) for OC lesion scores are shown in Figure 1. SNP exhibiting the most of significant association were located on SSC14. In fact, 8 SNPs of SSC 14 which were associated with OC lesion scores of CMH, CMF, and DEU, pointing to three regions. A single SNP (*ASGA0060524*) was found significantly associated with OC lesion scores at DEU that mapped to position 3.4 Mb. As for CMH, two SNPs (*M1GA0019792* and *M1GA0019802*) were identified and located on 144.9 and 145.2 Mb, respectively. Most interesting, significant associated with OC lesion scores of CMF were found for five SNPs (*ALGA0076446*, *MARC0098684*, *MARC00840086*, *MARC0093124*, and *ASGA0062794*) at 25.4 and between 36.1 and 38.2 Mb. In addition, there were 11 significantly associated SNPs which located on SSC3, 5, 8, 10, and 18 (Table 3). Genotype distributions for all significant SNPs in this study are provided in Table S1 in Supplementary Material.

**Table 3:** **SNPs associated with OC lesions scores according to GWAS**.

SNP	Trait	SSC	SNP location (bp)	*p*-value	*q*-value	OR	95% CI	Gene nearby	SSC	Gene location (bp)
*ALGA0076446*	CMF	14	25,436,055	2.31E−05	0.05	0.47	0.23–0.94	*TMEM132D*	14	25,391,692
*MARC0098684*	CMF	14	36,101,763	2.70E−05	0.05	0.45	0.22–0.94	*C12orf49*	14	35,976,703
*MARC0084086*	CMF	14	36,142,739	2.70E−05	0.05	0.45	0.22–0.94	*C12orf49*	14	35,976,703
*MARC0093124*	CMF	14	36,946,375	4.94E−05	0.09	0.46	0.22–0.97	*MED13L*	14	36,579,069
*ASGA0062794*	CMF	14	38,228,607	4.16E−05	0.08	0.45	0.21–0.96	*TBX3*, *TBX5*	14	37,979,782; 38,174,956
*MARC0017670*	CMH	3	63,326,409	4.80E−05	0.08	0.31	0.10–0.95	*MOB1A*	3	63,374,263
*H3GA0025124*	CMH	8	74,536,593	4.80E−05	0.08	0.31	0.10–0.95	*TBC1D9*	8	74,529,483
*M1GA0019792*	CMH	14	144,905,832	1.77E−06	0.00	0.28	0.10–0.79	*PTPRE*	14	144,447,776
*M1GA0019802*	CMH	14	145,226,743	4.86E−05	0.08	0.34	0.12–0.96	*MGMT*	14	145,738,712
*MARC0007896*	CMH	18	15,776,737	2.96E−05	0.06	0.35	0.13–0.93	*MKLN1*	18	15,657,789
*MARC0024273*	CMH	18	7,491,971	2.96E−05	0.06	0.35	0.13–0.93	*BRAF*	18	7,416,343
*MARC0069757*	DEU	5	83,513,211	4.57E−05	0.09	1.95	1.03–3.67	*NDUFA12*	5	83,512,621
*ASGA0060524*	DEU	14	3,467,306	2.12E−05	0.04	1.99	1.06–3.75	*SPTLC1*	14	3,487,545
*ALGA0105690*	DEU	18	2,442,820	4.06E−05	0.08	2.79	1.05–7.36	*DPP6*	18	2,430,867
*H3GA0010162*	OCcat	3	90,049,785	4.42E−05	0.08	0.50	0.25–0.98	*PLEKHH2*	3	90,049,914
*MARC0069757*	OCcat	5	83,513,211	4.79E−06	0.01	0.42	0.20–0.88	*NDUFA12*	5	83,512,621
*M1GA0007707*	OCcat	5	16,709,358	9.11E−06	0.02	2.29	1.11–4.75	*KRT8*	5	16,714,898
*MARC0100227*	OCcat	8	34,181,277	2.67E−05	0.05	0.45	0.22–0.94	*RASL11B*	8	34,044,032
*ASGA0083649*	OCcat	10	11,773,268	2.80E−05	0.05	2.45	1.07–5.65	*BROX*	10	11,596,786

*CMF, condylus medialis femoris; CMH, medial part of condyles humeri; DEU, distal epiphyseal cartilage of ulna; OCcat, category of animals with either high (OCsum ≥ 12) or low (OCsum ≤ 8) OCsum*.

**Figure 2:**
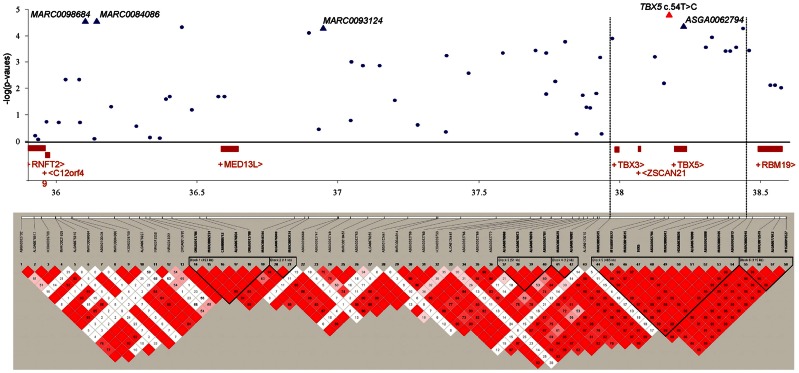
**Significance of association of SNPs of SSC14, 36.0–38.5 Mb, including *TBX5* c.54T > C with OC lesion score at CMF, regional assignment of SNPs and genes, and LD blocks**. Top: *p*-values (−log10 transformed) on the vertical axis are plotted against the position of SNPs on the horizontal axis. Bottom: linkage disequilibrium pattern in the chromosomal region. The scale of correlation coefficient varies from red to white (high to low).

We investigated the region between 36.0 and 38.5 Mb on SSC14 where multiple significant SNPs were located, that were associated with OC lesion scores at CMF. LD was calculated between the SNPs in this region across 2.5 Mb. Figure 2 shows the LD blocks of the region between 36.0 and 38.5 Mb on SSC14. Six discrete LD blocks were identified. The SNP *ASGA0062794* was assigned to a separate haplotype block spanning 465 kb. Gene annotation of this region showed two candidate genes within this haplotype block upstream of *ASGA0062794*, T-box transcription factors 3 and 5, *TBX3* and *TBX5*. The *TBX5* gene was located approximately 5.5 kb upstream of *ASGA0062794*. However, *TBX3* was located in substantial larger distance (237 kb) to *ASGA0062794*. Therefore, the *TBX3* and *TBX5* gene were selected to screen of sequence variation and to assess LD with the significant SNP-markers and association with OC in the same population used for the GWAS and LD study.

**Figure A2:**
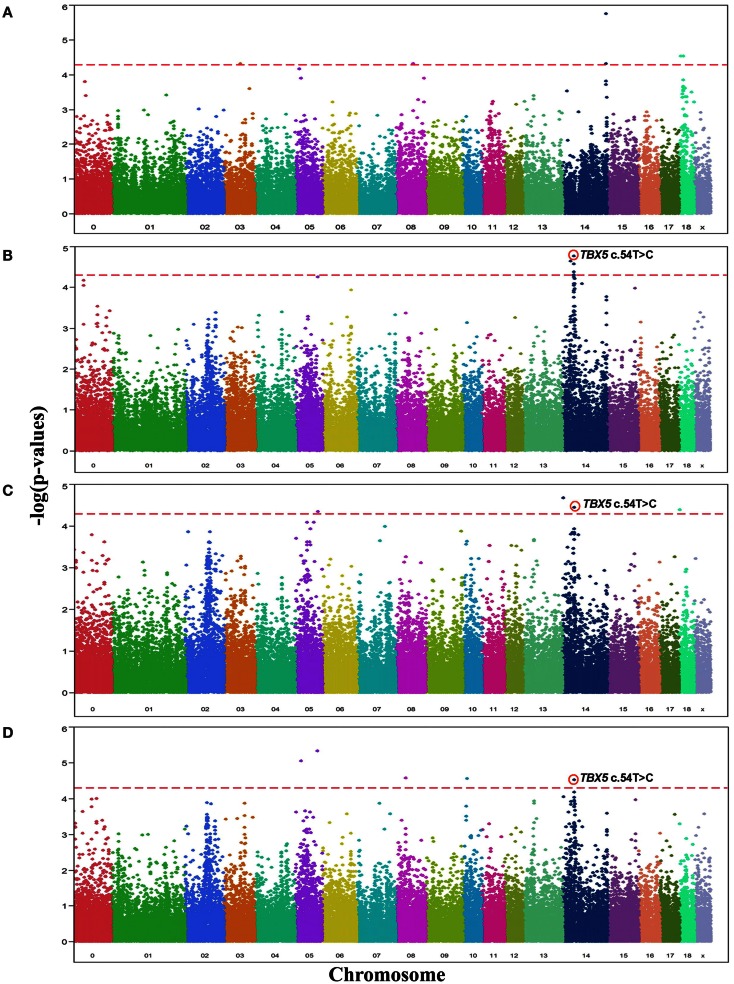
**Manhattan plots displaying the significance of associations of SNPs and in particular the SNP TBX5 c.54T > C with OC lesion scores at (A) medial part of condylus humeri (CMH), (B) condylus medialis femoris (CMF), (C) distal epiphyseal cartilage of ulna (DEU), and (D) category of OC (OCcat)**. The *p*-values (−log10 transformed) on the vertical axis are plotted against the genomic position of SNPs on the horizontal axis. The horizontal dashed line indicates the genome-wide significance level (*q*-value ≤ 0.1).

**Figure A3:**
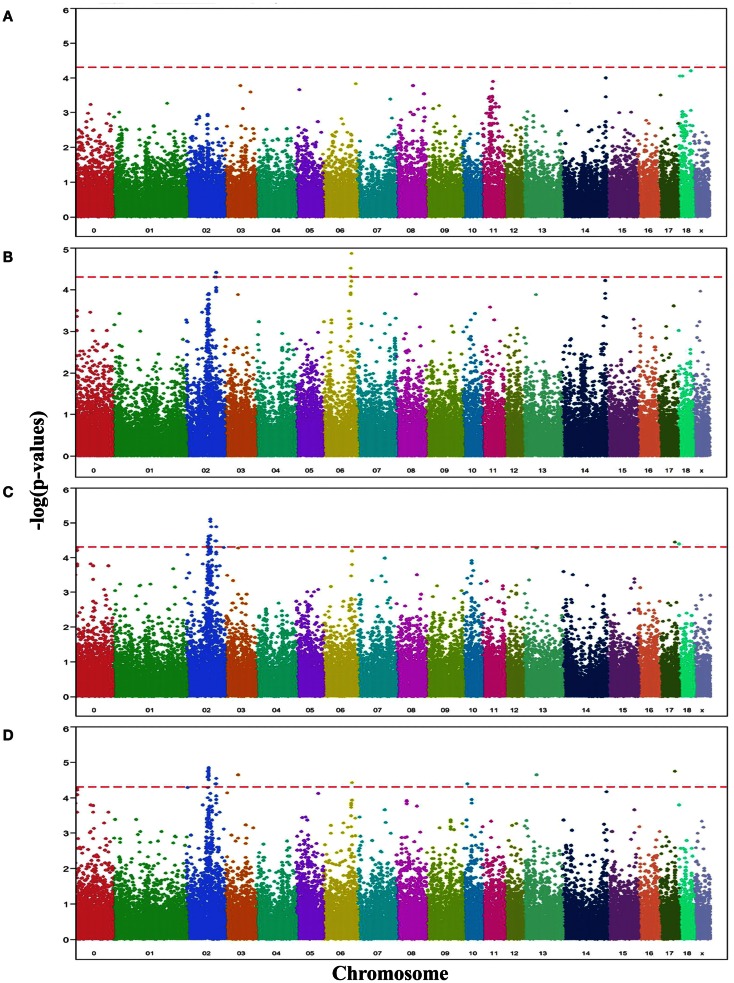
**Manhattan plots displaying the significance of associations of SNPs when using the SNP TBX5 c.54T > C as a fixed effect with OC lesion scores at (A) medial part of condylus humeri (CMH), (B) condylus medialis femoris (CMF), (C) distal epiphyseal cartilage of ulna (DEU), and (D) category of OC (OCcat)**. The *p*-values (−log10 transformed) on the vertical axis are plotted against the genomic position of SNPs on the horizontal axis. The horizontal dashed line indicates the genome-wide significance level (*q*-value ≤ 0.1).

**Figure A4:**
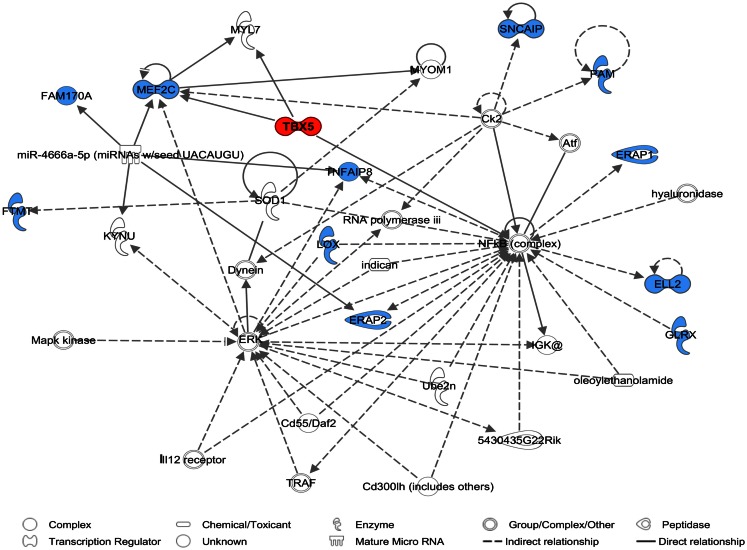
**A molecular network of the TBX5 gene with genes assigned to SSC2 87.3–115.0 Mb marked in blue derived by Ingenuity Pathway Analysis (IPA)**. The network belongs to IP biofunctions embryonic development, organismal development, and skeletal and muscular system development and function.

Comparative sequencing of the coding region of *TBX3* revealed no polymorphisms. In the *TBX5* gene, a single synonymous SNP was detected at position 54 relative to the start codon (c.54T > C) of *TBX5*. The SNP *TBX5* c.54T > C showed a high LD of *r^2^* = 0.96 and *D*′ = 1.0 with *ASGA0062794*. The single-marker association analysis between *TBX5* c.54T > C with OC lesion scores is shown in Table 4. Significant additive and dominant effects were found for OC lesion scores at CMF (*p* < 0.0001, OR = 0.40, 95% CI = 0.26–0.61), DEU (*p* < 0.0005, OR = 0.45, 95% CI = 0.31–0.66), and OCcat (*p* < 0.0005, OR = 2.49, 95% CI = 1.63–3.82). Moreover, when integrating the SNP *TBX5* c.54T > C in the GWAS together with the 48,716 SNPs of the SNPchip, it showed highest statistical significance of association with CMF and DEU among the markers of the haplotype block and even reached the significance threshold for the trait OCcat (see Figure A2 in Appendix). In addition, using the *TBX5* c.54T > C genotypes as a fixed effect in the statistical model revealed a drop of the −log10(*p*-value) of all markers in the region below the significance threshold indicating that the SNP *TBX5* c.54T > C explains much of the variation in OC lesion scores depending on this genomic region (see Figure A3 in Appendix). Interestingly, at the same time a number of SNPs located on SSC2 between 87.3 and 115.0 Mb become significant, when treating *TBX5* c.54T > C as a fixed effect (Table S2 in Supplementary Material). This may be simply due to the reduction of residual variance. However, it might also be a hint for epistatic interaction between *TBX5* and a gene located in the region on SSC2. Indeed, Ingenuity Pathway Analysis revealed a network of indirect and direct effects between *TBX5* and a number of genes all being located between 87.3 and 115 Mb on SSC2 (Figure A4 in Appendix). These functionally linked genes are: *MEF2C* (myocyte enhancer factor 2 C), *ELL2* (elongation factor, RNA polymerase II, 2), *GLRX* [glutaredoxin (thioltransferase)], *ERAP1* (endoplasmic reticulum aminopeptidase 1), *ERAP2* (endoplasmic reticulum aminopeptidase 2), *PAM* (peptidylglycine alpha-amidating monooxygenase), *TNFAIP8* (tumor necrosis factor, alpha-induced protein 8), *FAM170A* (family with sequence similarity 170, member A), *FTMT* (ferritin mitochondrial), *LOX* (lysyl oxidase), and *SNCAIP* (synuclein, alpha interacting protein). The logistic regression model complemented with an appropriate interaction term of *TBX5* c.54T > C and SNPs on SSC2 revealed pairs of SNPs with significant interaction effects (Table S3 in Supplementary Material).

**Table 4:** **Association of *TBX5* c.54T > C with OC lesion scores**.

Traits	Additive model		*p*-value	Dominant model		*p*-value
	OR	95%CI		OR	95%CI	
CMH	0.72	0.443–1.172	0.1862	0.77	0.407–1.437	0.4054
CMF	0.40	0.264–0.606	<0.0001	0.31	0.177–0.532	<0.0001
DEU	0.45	0.313–0.659	<0.0001	0.41	0.254–0.652	0.0002
OCcat	2.49	1.627–3.818	<0.0001	2.64	1.566–4.449	0.0003

*CMH, medial part of condyles humeri; CMF, condylus medialis femoris; DEU, distal epiphyseal cartilage of ulna; OCcat, category of animals with either high (OCsum ≥ 12) or low (OCsum ≤ 8) OCsum*.

**Figure A5:**
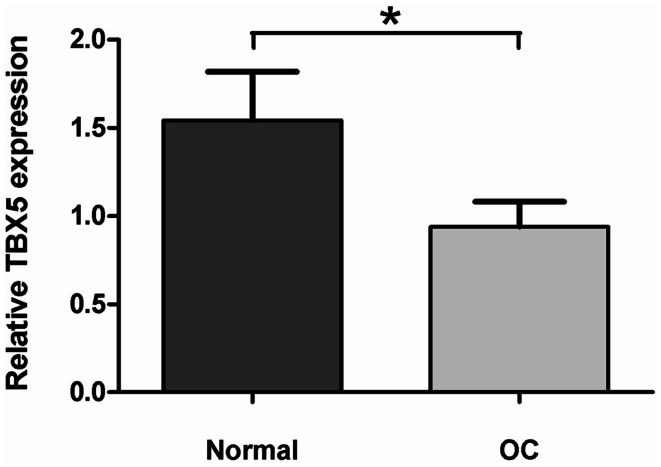
**TBX5 expression in normal and osteochondrosis (OC) lesion of articular cartilage tissues (*n* = 8)**. Relative TBX5 expression was determined by the 2^−ΔΔ^ method (Livak and Schmittgen, 2001). Data represents mean ± SEM. *p < 0.05.

Because the screening for polymorphisms of *TBX5* did not reveal any SNP leading to amino acid exchange we performed qPCR of CMF cartilage samples of divergent sib pairs with either low or high OC lesion scores in order to test the hypothesis that *TBX5* may exhibit a regulatory polymorphism in LD with the SNP *TBX5* c.54T > C. Significant differences were found (*p* < 0.05) with 1.63-fold higher transcript abundance in the normal cartilages compared with OC cartilages (Figure A5 in Appendix).

## Discussion

In this study, markers were detected that were significantly associated with OC lesions scores using the GWAS approach. Moreover, we confirmed the association of a positional candidate gene which might be involved in liability to develop OC lesions.

The GWA analyses revealed 19 SNPs significantly associated with either one or more of the traits related to OC (CMH, CMF, DUE, and OCcat). Among these, 12 SNPs were located in previously reported QTL regions for leg weakness and OC related traits in pigs on SSC3, 5, 14, and 18.

The most prominent region harboring SNPs associated with OC lesion scores was located on SSC14 that corresponded to a QTL region for leg conformation (Lee et al., 2003). Gene annotation of this region revealed *TBX3* (T-box 3) and *TBX5* (T-box 5) as candidate genes for the liability to develop OC lesions. *TBX3* and *TBX5* belong to the T-box gene family that were demonstrated to function as transcriptional activators and repressors (Wilson and Conlon, 2002).

Additionally, one region at 144.9–145.2 Mb of SSC14 encompassed two SNPs, and it contains the *PTPRE* (protein tyrosine phosphatase receptor E) and the *MGMT* (O-6-methyguanine-DNA methyltransferase) gene, respectively. *PTPRE* plays a major role in regulating bone structure and metabolism (Schiller and Mauro, 2005). Chiusaroli et al. (2004) showed that *PTPRE* as a phosphatase is required for optimal structure, subcellular organization and function of osteoclasts *in vivo* and *in vitro*.

On SSC3, we detected two significant SNPs that were 0.05 Mb upstream of the *MOB1A* (MOB kinase activity 1A) gene and 0.13 kb downstream of the *PLEKHH2* (pleckstrin homology domain containing, family H member 2) gene, respectively. There are no obvious functional links between *MOB1A*, or *PLEKHH2* and OC lesions. However, the region (63.3–90.0 Mb) is consistent with the QTL for leg weakness and OC related traits of previous studies (Lee et al., 2003; Uemoto et al., 2010).

On SSC5, one region located at 16.7 Mb showed an association with OC lesions. This region harbors *KRT8* (keratin 8) which may play a role in the endothelial inflammatory disease, as atherosclerosis. The mapping site of *KRT8* on the porcine SSC5 matches in a QTL region for OC lesions of fissure between cartilage and bone and OC (Andersson-Eklund et al., 2000; Christensen et al., 2010). We have previously shown an association of the SNP *KRT8* g.8,039G > A with OC score at CMH and bone mineralization (Rangkasenee et al., 2013).

On SSC18, we detected two significant SNPs showing association with OC lesion at CMH which are located at 7.49 and 15.77 Mb in the *BRAF* (v-raf murine sarcoma viral oncogene homolog B1) and the *MKLN1* (muskelin 1) gene, respectively. These sites match a QTL for front leg conformation detected by (Lee et al., 2003). BRAF is expressed in proliferating chondrocytes, however, it was shown that *BRAF* is dispensable for endochondral bone development as indicated by conditional removal from cartilage which did not affect chondrocyte proliferation and maturation (Provot et al., 2008).

We considered *TBX5* as a strong candidate gene and identified a polymorphism in exon 1 being associated with OC lesion scores. *TBX5* has been described as DNA-binding transcriptional regulators that plays diverse roles during differentiation and development (Papaioannou and Silver, 1998; Wilson and Conlon, 2002). In this study, we detected the a novel synonymous SNP (c.54T > C) within the N-terminal domain of *TBX5* gene that is involved in the synergistic regulation of transcriptional activity of the downstream target gene (Fan et al., 2003). Neither other polymorphisms were detected in the coding region nor were previously described synonymous SNPs segregating among the animals. This might indicate that regulatory SNPs are involved in the effects of *TBX5*. This hypothesis is supported by the significant differences in the abundance of *TBX5* transcripts in cartilage of the CMF with either low or high OC lesion scores.

Interestingly, *TBX5* exerts its role in bone growth and maturation by controlling gene expression of gap junction protein, alpha 5, 40 kDa, [*GJA5*, also named connexin 40 (*CX40*)] which was identified as a target gene of *TBX5* and harbors binding site for transcriptional factor of *TBX5* in its promoter (Bruneau et al., 2001; Pizard et al., 2005). Mutations in either *TBX5* or *GJA5* genes reducing *GJA5* expression and result in anomalous joint formation and erroneous bone length (Pizard et al., 2005). Moreover, Fang et al. (2012) demonstrated that *GJA5* is necessary for post-ischemic limb survival and reperfusion in a model of vascular obstruction. *GJA5* deficient mice fail to recover from ischemic lesions that lead to necrosis after surgery at hind limb. In this context, it is noteworthy that failure of blood supply to growing cartilage was found to contribute to OC development (Ytrehus et al., 2004a,b). Thus, *TBX5* may contribute to the liability for osteochondral defects via its regulatory impact on *GJA5*. Porcine *GJA5* is located on SSC4 at 104 Mb in a region where there is no indication of polymorphisms associated with OC lesion scores neither from previous QTL studies nor this GWAS.

*MEF2C* is among the genes located on SSC2 in the region that becomes significantly associated with OC lesion scores, if *TBX5* is treated as a fixed factor. *TBX5* and *MEF2C* were shown to activate *MYH6* synergistically during heart development (Ghosh et al., 2009). *MEF2C* is known as a transcriptional factor that plays a critical role in involved in muscle and cardiovascular development. Interestingly, it was also shown to affect bone development by modulating chondrocyte hypertrophy and cartilage angiogenesis (Arnold et al., 2007). So there is a line of evidence for epistatic interaction of *TBX5* and *MEF2C* that may relate to osteochondral defects. The functional links support deficiency of blood supply to growth cartilage as being fundamental for the initiation of OC. However, the interaction between *TBX5* and *ASGA0010975* (SNP nearby *MEF2C* gene) was not significant associated with OC lesion (*p* = 0.18).

Taken together, previous reports suggested that there is functional evidence for the association of *TBX5* with OC lesion scores. This study provides further positional and genetic-statistical evidence for the candidacy of *TBX5*. However, beside *TBX5* also two other genes (*TBX3* and *ZSCAN21*) reside in the same haplotype block and may potentially be responsible for the association with OC lesions. *TBX3* is expressed in the anterior and posterior margins of both fore limb and hind limb buds. The posterior expression of *TBX3* is crucial for development of the more distal limb elements (Wilson and Conlon, 2002). The function of *ZSCAN21* is currently unknown.

In summary, we identified *TBX5* as the positional candidate genes located in a block of markers in high LD. In fact, there is multiple evidence for the role of *TBX5* for the liability to develop OC lesions including functional aspects and genetic-statistical issues obtained here: (1) the high LD of *TBX5* c.54T > C with the significant SNP-markers at 36.0–38.5 Mb of SSC14, (2) its association with OC lesion scores confirmed by single-marker analysis, (3) the superior association of *TBX5* c.54T > C when included in the genome scan, and (4) the LOD-drop when fitting the GWAS model using the *TBX5* c.54T > C genotype as an additional fixed. The results imply that the association signal obtained on SCC14 is largely attributable to *TBX5* c.54T > C, or other as-yet-unspecified polymorphisms in LD that most likely affect the expression of *TBX5*. The functional role of *TBX* in synergy with *GJA5* and *MEF2C* promote deficiency of blood supply to growth cartilage as being fundamental for the initiation of OC.

## Conflict of Interest Statement

The authors declare that the research was conducted in the absence of any commercial or financial relationships that could be construed as a potential conflict of interest.

## Supplementary Material

The Supplementary Material for this article can be found online at http://www.frontiersin.org/Livestock_Genomics/10.3389/fgene.2013.00078/abstract

## Footnotes

^1^http://www.animalgenome.org/cgi-bin/QTLdb/SS/summary

^2^http://www.ensembl.org/index.html

^3^http://www.ncbi.nlm.gov/mapview/

^4^http://www.animalgenome.org/cgi-bin/QTLdb/SS/index
