# Supplementary material for: Genome-Wide Association Identifies TBX5 as Candidate Gene for Osteochondrosis Providing a Functional Link to Cartilage Perfusion as Initial Factor
Source: Front Genet. 2013 May 10;4:78. doi: 10.3389/fgene.2013.00078 (PMC3650520; doi:10.3389/fgene.2013.00078)
Supplement: Supplementary file 1 [file 44377_Wimmers_DataSheet1.PDF]

<u>Supplementary Table 1</u> Genotype distribution of SNPs associated with osteochondrosis (OC) lesion in the Large White population

**Trait**	**SNP**	**Genotypes**	**OC lesion scores (n)**					
			**1**	**2**	**3**	**4**	**5**	**6**
CMF	*ALGA0076446*	AG	82	40	9	1		
		GG	83	30	2	0		
		AA	22	16	10	1		
	MARC0098684	TT	110	28	9	1		
		AT	66	46	8	1		
		AA	11	12	4	0		
	MARC0084086	GG	110	28	9	1		
		AG	66	46	8	1		
		AA	11	12	4	0		
	MARC0093124	AA	110	28	8	1		
		AC	66	48	9	1		
		CC	10	10	3	0		
	ASGA0062794	AA	108	29	6	1		
		AG	69	46	13	1		
		GG	10	11	2	0		
	TBX5 c.54T>C	CT	62	42	10	1		
		TT	87	21	4	1		
		CC	9	10	3	0		
CMH	*MARC0017670*	AA	203	22	15			
		AG	32	8	11			
		GG	2	1	2			
	H3GA0025124	AA	203	22	15			
		AG	32	8	11			
		GG	2	1	2			
	M1GA0019792	AA	191	14	17			
		AG	45	16	6			
		GG	1	1	5			
	M1GA0019802	AA	184	15	16			
		AG	51	15	8			
		GG	2	1	4			
	MARC0007896	AG	133	23	15			
		AA	75	2	3			
		GG	29	6	10			
	MARC0024273	AC	133	23	13			
		AA	75	2	3			
		CC	29	6	10			

DEU	*MARC0069757*	AC	17	72	3	20	28	2
		CC	6	33	11	22	34	0
		AA	8	27	1	5	7	0
	ASGA0060524	AG	14	74	6	28	34	1
		GG	4	27	6	14	26	1
		AA	13	31	3	5	9	0
	ALGA0105690	GG	16	97	14	42	59	1
		AG	13	34	1	5	9	1
		AA	2	1	0	0	1	0
			**Low**	**High**				
OCcat	*H3GA0010162*	AG	81	60				
		GG	32	54				
		AA	48	21				
	MARC0069757	AC	88	54				
		CC	38	68				
		AA	35	13				
	M1GA0007707	AG	83	73				
		AA	60	24				
		GG	18	38				
	MARC0100227	GG	64	81				
		AG	35	17				
		AA	35	11				
	ASGA0083649	GG	99	57				
		AG	61	61				
		AA	1	17				

CMF, condylus medialis femoris; CMH, medial part of condyles humeri; DEU, distal epiphyseal cartilage of ulna; OCcat, category of animals with either high (OCsum $\geq$ 12) or low (OCsum $\leq$ 8) OCsum

<u>Supplementary Table 2</u> SNPs on SSC2 showing significant association with OC lesion scores after treating *TBX5* c.54T>C as a fixed effect

SNP	Trait	SNP location (bp)	p-value	q-value	OR	95% CI	Gene nearby
ASGA0010975	OCcat	87,321,358	2.61E-05	0.10	0.39	0.16 – 0.93	*TMEM161B, MEF2C, CETN3, POLR3G, GPR98, ARRDC*
	DEU		4.66E-05	0.10	0.47	0.23 – 0.97	
ASGA0010980	OCcat	87,473,211	1.8E-05	0.10	0.38	0.16 – 0.91	
	DEU		3.66E-05	0.10	0.46	0.22 – 0.96	
MARC0019188	OCcat	87,514,617	2.61E-05	0.10	0.39	0.16 – 0.93	
	DEU		4.66E-05	0.10	0.47	0.23 – 0.97	
ALGA0014591	OCcat	87,841,659	2.69E-05	0.10	0.39	0.16 – 0.93	
	DEU		5.03E-05	0.10	0.47	0.22 – 0.97	
H3GA0007224	OCcat	87,876,494	2.69E-05	0.10	0.39	0.16 – 0.93	
	DEU		5.03E-05	0.10	0.47	0.22 – 0.97	
MARC0028129	OCcat	87,898,409	2.69E-05	0.10	0.39	0.16 – 0.93	
	DEU		5.03E-05	0.10	0.47	0.22 – 0.97	
ALGA0014601	OCcat	87,999,677	2.69E-05	0.10	0.39	0.16 – 0.93	
	DEU		5.03E-05	0.10	0.47	0.22 – 0.97	
ALGA0014610	OCcat	88,692,061	2.03E-05	0.10	0.39	0.16 – 0.93	
	DEU		3.21E-05	0.10	0.46	0.22 – 0.95	
H3GA0007228	OCcat	88,833,650	1.41E-05	0.10	0.37	0.15 – 0.90	
	DEU		2.48E-05	0.10	0.45	0.22 – 0.94	
ALGA0014617	OCcat	88,886,145	2.47E-05	0.10	0.39	0.16 – 0.93	
DRGA0003207	OCcat	88,934,240	2.47E-05	0.10	0.39	0.16 – 0.93	
DRGA0003209	OCcat	88,957,812	1.71E-05	0.10	0.38	0.16 – 0.91	
	DEU		4.05E-05	0.10	0.46	0.22 – 0.96	
DRGA0003215	OCcat	89,314,668	1.55E-05	0.10	0.38	0.16 – 0.91	
	DEU		2.93E-05	0.10	0.46	0.22 – 0.95	

MARC0035937	OCcat	89,433,132	1.55E-05	0.10	0.38	0.16 – 0.91	*NR2F1, FAM172A, MCTP1, TTC37, RHOBTB3, ELL2, GLRX, PCSK1, CAST*
	DEU		2.93E-05	0.10	0.46	0.22 – 0.95	
DRGA0003220	OCcat	89,598,528	3.20E-05	0.10	0.39	0.17 – 0.94	
DRGA0003201	OCcat	89,717,832	1.41E-05	0.10	0.37	0.15 – 0.90	
	DEU		2.48E-05	0.10	0.45	0.22 – 0.94	
DRGA0003223	OCcat	89,744,691	2.26E-05	0.10	0.39	0.16 – 0.93	
ALGA0114318	OCcat	90,298,885	3.20E-05	0.10	0.39	0.17 – 0.94	
MARC0013344	OCcat	90,428,562	2.26E-05	0.10	0.39	0.16 – 0.93	
DIAS0004805	OCcat	90,489,177	2.26E-05	0.10	0.39	0.16 – 0.93	
	DEU		5.0E-05	0.10	0.47	0.23 – 0.97	
ASGA0011092	DEU	94,385,370	2.98E-05	0.10	2.21	1.06 – 0.46	*ERAP1, ERAP2, LNPEP, LIX1, RGMB, CHD1, FAM174A, ST8SIA4*
ALGA0107105	DEU	95,247,092	9.16E-06	0.10	2.31	1.11 – 4.83	
DRGA0003296	DEU	95,371,839	7.88E-06	0.10	2.31	1.11 – 4.80	
ALGA0106053	DEU	95,397,739	9.16E-06	0.10	2.31	1.11 – 4.83	
H3GA0007290	DEU	95,462,189	9.16E-06	0.10	2.31	1.11 – 4.83	
DIAS0004763	DEU	95,482,507	9.16E-06	0.10	2.31	1.11 – 4.83	
ALGA0014815	DEU	95,534,140	2.40E-05	0.10	2.28	1.07 – 4.87	
ALGA0014821	DEU	95,593,410	2.44E-05	0.10	2.28	1.07 – 4.86	
ALGA0014907	DEU	97,635,902	1.31E-05	0.10	2.29	1.11 – 4.83	*SLCO4C1, SLCO6A1, PAM, GIN1, NUDT12*
ALGA0014911	DEU	97,662,994	4.97E-05	0.10	2.17	1.03 – 4.55	
MARC0017383	DEU	111,000,000	3.39E-05	0.10	0.48	0.24 – 0.96	*DTWD2, DMXL1, TNFAIP8, FAM170A, PRR16, FTMT, LOX, ZNF474, SNCAIP, CEP120, CSNK1G3, ZNF608*
	OCcat		4.14E-05	0.10	0.42	0.19 – 0.96	
	CMF		5.04E-05	0.10	0.44	0.20 – 0.97	
ALGA0015731	DEU	114,800,000	1.33E-05	0.10	0.46	0.23 – 0.92	
	OCcat		2.87E-05	0.10	0.41	0.18 – 0.94	
	CMF		3.87E-05	0.10	0.43	0.19 – 0.96	
ASGA0011739	CMF	115,000,000	5.04E-05	0.10	0.44	0.20 – 0.97	
	DEU		3.39E-05	0.10	0.48	0.24 – 0.96	
	OCcat		4.14E-05	0.10	0.42	0.19 – 0.96	

<u>Supplementary Table 3</u> Significant interactions of *TBX5* c.54T>C and SNPs located on SSC2 that showing significant association with OC lesion scores after treating *TBX5* c.54T>C as a fixed effect

**SNP pair interaction**	**SNP location (SSC2; bp)**	**Trait associated**	**p-value**	**Gene nearby**
TBX5 × ASGA0011092	94,385,370	DEU	0.0247	*ERAP1*, *ERAP2*, *LNPEP*, *LIX1*, *RGMB*, *CHD1*, *FAM174A*, *ST8SIA4*
TBX5 × ALGA0107105	95,247,092	DEU	0.0418	
TBX5 × DRGA0003296	95,371,839	DEU	0.0447	
TBX5 × ALGA0106053	95,397,739	DEU	0.0418	
TBX5 × H3GA0007290	95,462,189	DEU	0.0418	
TBX5 × DIAS0004763	95,482,507	DEU	0.0418	
TBX5 × ALGA0014815	95,534,140	DEU	0.0181	
TBX5 × ALGA0014821	95,593,410	DEU	0.0190	
TBX5 × ALGA0014907	97,635,902	DEU	0.0253	*SLCO4C1*, *SLCO6A1*, *PAM*, *GIN1*, *NUDT12*
TBX5 × ALGA0014911	97,662,994	DEU	0.0483	
TBX5 × MARC0017383	111,000,000	CMF	0.0065	*DTWD2*, *DMXL1*, *TNFAIP8*, *FAM170A*, *PRR16*, *FTMT*, *LOX*, *ZNF474*, *SNCAIP*, *CEP120*, *CSNK1G3*, *ZNF608*
TBX5 × ALGA0015731	114,845,161	CMF	0.0060	
TBX5 × ASGA0011739	114,990,969	CMF	0.0065	
